# More than Addiction—The Nucleus Accumbens Contribution to Development of Mental Disorders and Neurodegenerative Diseases

**DOI:** 10.3390/ijms23052618

**Published:** 2022-02-27

**Authors:** Martyna Bayassi-Jakowicka, Grazyna Lietzau, Ewelina Czuba, Cesare Patrone, Przemysław Kowiański

**Affiliations:** 1 Division of Anatomy and Neurobiology, Faculty of Medicine, Medical University of Gdańsk, Dębinki 1, 80-211 Gdansk, Poland; mbayassi@gumed.edu.pl (M.B.-J.); ewelina.czuba@gumed.edu.pl (E.C.); 2 Department of Clinical Science and Education, Södersjukhuset, Internal Medicine, Karolinska Institutet, Sjukhusbacken 17, 11883 Stockholm, Sweden; cesare.patrone@ki.se

**Keywords:** addiction, Alzheimer’s disease, depression, Huntington’s disease, nucleus accumbens, Parkinson’s disease, schizophrenia

## Abstract

Stress and negative emotions evoked by social relationships and working conditions, frequently accompanied by the consumption of addictive substances, and metabolic and/or genetic predispositions, negatively affect brain function. One of the affected structures is nucleus accumbens (NAc). Although its function is commonly known to be associated with brain reward responses and addiction, a growing body of evidence also suggests its role in some mental disorders, such as depression and schizophrenia, as well as neurodegenerative diseases, such as Alzheimer’s, Huntington’s, and Parkinson’s. This may result from disintegration of the extensive connections based on numerous neurotransmitter systems, as well as impairment of some neuroplasticity mechanisms in the NAc. The consequences of NAc lesions are both morphological and functional. They include changes in the NAc’s volume, cell number, modifications of the neuronal dendritic tree and dendritic spines, and changes in the number of synapses. Alterations in the synaptic plasticity affect the efficiency of synaptic transmission. Modification of the number and structure of the receptors affects signaling pathways, the content of neuromodulators (e.g., BDNF) and transcription factors (e.g., pCREB, DeltaFosB, NFκB), and gene expression. Interestingly, changes in the NAc often have a different character and intensity compared to the changes observed in the other parts of the basal ganglia, in particular the dorsal striatum. In this review, we highlight the role of the NAc in various pathological processes in the context of its structural and functional damage, impaired connections with the other brain areas cooperating within functional systems, and progression of the pathological processes.

## 1. Introduction

Striving for the goals of a professional career, higher social and/or economic status, often results in stress and negative emotions, deteriorating self-esteem, mental functions, and behavioral reactions. All these factors could enhance the development of some mental disorders such as schizophrenia and depression. When coexisting with genetic predispositions, metabolic changes, and/or environmental factors having a negative effect on the CNS, they can also augment the probability of development of some neurodegenerative diseases such as Alzheimer’s (AD), Huntington’s (HD), and Parkinson’s disease (PD). Accumulating data suggests that at the pathomorphological basis of these processes are changes in the limbic system [1]. One of the areas affected by the negative stimuli is the nucleus accumbens (NAc), a small region in the forebrain localized in the ventral part of the striatum, considered a part of the basal ganglia and the brain reward system (BRS). This brain area has an integrative function upon limbic, motor, sensory, and vegetative systems [1]. The NAc’s connections with cortical areas, the basal ganglia, diencephalon, and the brain stem involve all major neurotransmitter systems. This enabled the development of precise functional synaptic plasticity mechanisms, relying on molecular processes which enable the most efficient impulse conduction, determining the NAc role in the physiological conditions. However, this complex system is susceptible to alterations resulting from action of the external pathological stimuli or being a consequence of the disturbed metabolic processes, signaling pathways or genetic mutations [2,3,4,5,6]. These changes could lie at the basis of not only addiction, but also some psychiatric disorders and neurodegenerative diseases. Despite the extensive research on the mechanisms leading to the development of such CNS pathologies as schizophrenia, depression, and AD, our understanding of their molecular and structural basis is still incomplete.

In our previous publication [7], we focused on the specific pattern of connections, neuroplasticity processes, and the neurotransmitter systems responsible for the complex function of the NAc in the CNS. In this review, we summarize the current state of knowledge on molecular mechanisms and morphological changes underlying the NAc dysfunction in addiction, selected mental disorders, and neurodegenerative diseases. We also characterize the functional relationships between the NAc and the other brain regions, which are important for the development and progression of the CNS diseases. The role of the NAc in the development of pathological symptoms is emphasized.

## 2. Addiction-Induced Short- and Long-Term Changes in Synaptic Plasticity Affect Molecular Signaling and Spino-Dendritic Morphology of the Accumbal Neurons

Addiction relies on compulsive striving for the consumption of certain substances or performing specific activities of rewarding value despite adverse, negative consequences [2,8]. Many authors emphasize a significant role of environmental pressure, genetic conditions, and life experiences in the development of addiction [8,9,10]. Important features of addiction are drug or reward craving, inadequate emotional responses, failure to recognize the reasons affecting behavior, and an inability to control disturbed behavior. In the light of current views, the concept of addiction is closely related to the advanced form of substance-use disorder [10,11,12]. Commonly used stimulants, such as alcohol, nicotine, drugs, and hallucinogens have addictive properties. Intake of addictive substances induces changes in the functioning of the NAc and the disruption of plasticity processes within its synaptic system. However, the mechanisms of these negative changes are different in each case and not completely disclosed.

### 2.1. Alcohol

**Figure 1:**
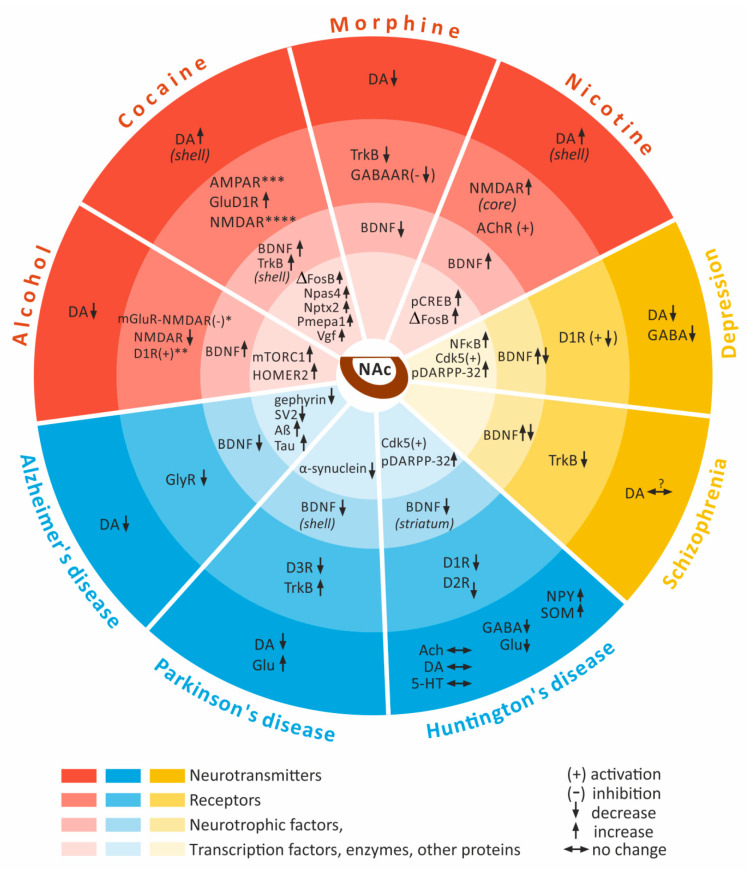
Psychostimulant-induced and related mental illnesses and neurodegenerative diseases changes to the expression and activation level of different neuronal factors in the nucleus accumbens. * inhibition of group I mGluR-mediated potentiation of NMDAR, ** enhancement of excitatory synaptic transmission onto D1R+ neurons, *** long-term changes in AMPAR-dependent synaptic efficacy, **** impairment of NMDAR-dependent long-term potentiation and long-term depression at glutamatergic synapses. **Abbreviations: Aß**—amyloid ß; **ACh**—acetylcholine; **AChR**—acetylcholine receptor; **AMPAR**—α-amino-3-hydroxy-5-methyl-4-isoxazolepropionic acid-type glutamate receptor; **BDNF**—brain-derived neurotrophic factor; **Cdk5**—cyclin-dependent kinase 5; **DA**—dopamine; **DARPP-32**—cAMP-regulated phosphoprotein 32; D1R—dopamine D1 receptor; **D2R**—dopamine D2 receptor; **D3R**—dopamin D3 receptor; **GABA**—γ-aminobutyric acid; **GABAAR γ**—aminobutyric acid type A receptor; **Glu**—glutamate; **GluD1R**—glutamate delta-1 receptor; **GlyR**—glycine receptor; **HOMER2**—Homer scaffolding protein 2; **mGluR**—metabotropic glutamate receptor; **mTOR1**—mechanistic target of rapamycin complex 1; **NFκB**—Nuclear factor kappa B; **NMDAR**—N-methyl-D-aspartate receptor; **Npas4**—neuronal PAS domain protein 4; **Nptx2**—neuronal pentraxin 2; **NPY**—neuropeptide Y; **pCREB**—phosphorylated adenosine 3′5′ cyclic monophosphate response element binding protein; **Pmepa1**—prostate transmembrane protein, androgen induced 1; **SOM**—somatostatin; **TrkB**—tropomyosin receptor kinase B; **5-HT**—serotonin.

Although alcohol is one of the widely used addictive substances with anxiolytic, mood-enhancing, and rewarding effects, it slows reaction times, causes motor incoordination, and impairs judgment abilities [13]. Its mechanism of action on synaptic plasticity remains poorly understood [2]. It has been suggested that alcohol consumption results in long-term potentiation (LTP) impairment in the NAc [14]. It may be a result of the changed interactions between the NMDARs and the group I metabotropic glutamatergic receptors (mGluRs) required for the development of LTP, as well as of the reduced dopamine (DA) availability (Figure 1). The disruption of the synaptic plasticity, being a result of the dysfunction of NMDARs in the NAc core, most likely leads to an increased tendency towards alcohol consumption. The decreased expression of NMDARs results in the development of behavioral sensitization to alcohol [15].

Another regulatory mechanism involves the dopaminergic D1 receptor (D1R)-containing GABA-ergic neurons in the NAc. Increase in the mTORC1 expression in the D1R-containing neurons in the NAc shell was already observed during the first alcohol intoxication [16]. This can explain the stimulating effect of alcohol on synaptic plasticity already during the first consumption. On the contrary, blocking mTORC1 expression resulted in reduced alcohol consumption during the first session.

Control of alcohol consumption is also related to regulation of the inhibitory effects of GABA-ergic neurons in the NAc by the other types of dopaminergic receptors. It has been recently shown that D3R genetic-driven enhancement of α6 GABAA subunit expression inhibits voluntary alcohol intake and increases GABA inhibition in the NAc [17].

Castelli and colleagues provided data connecting the expression of the Homer family of adaptor proteins with alcohol consumption [18]. In the alcohol stimulation model, they have shown that the expression of Homer 2 protein increases in the NAc along with repeated alcohol consumption. This suggests a role of this brain area in the alcohol-induced alterations of the synaptic plasticity mechanisms. However, the role of the Homer 2 protein in regulating addiction susceptibility remains unclear. Addressing this issue could help to detect molecular mediators responsible for the alcohol-related functional disorders. These mediators could be the potential therapeutic targets for future addiction treatment strategies.

Finally, changes in BDNF expression have been found in several brain areas associated with the development of addiction after heavy alcohol consumption [19]. However, the role of BDNF in the synaptic plasticity processes within the NAc initiated by alcohol consumption is not completely understood. Therefore, in order to develop more effective methods for treating alcohol addiction, the molecular bases of all above-presented the NAc-related processes should be further investigated.

Further studies on the influence of alcohol consumption on NAc should be focused on the role of neurotransmitter systems, such as the dopaminergic and glutamatergic, their receptors, neuroplasticity mechanisms in the NAc, and its connections with brain areas regulating emotional and motivational behavior.

### 2.2. Cocaine

Studies have shown that cocaine administration evokes an increase in DA content in the NAc shell (Figure 1) [20]. This suggests initiation of the addiction processes largely based on functional synaptic plasticity. Although mechanisms of these processes are only partially disclosed, some authors point to the complex nature of these phenomena [20,21]. Frequent exposure to cocaine affects AMPAR-mediated signaling and induces AMPAR-dependent long-term changes in synaptic efficacy within the NAc shell [21]. Results of animal studies showed that these changes depend on the level of AMPARs expression and the receptor’s subunit composition [21]. Furthermore, an increased expression of the orphan glutamate delta-1 receptor (GluD1R) has been reported in the NAc after cocaine administration [22]. GluD1R is a member of the ionotropic glutamate receptor family regarded as an important factor in the regulation of cocaine susceptibility. This takes place through changing composition of the NMDA receptors subunits (i.e., incorporation of GluN2B subunit) in the MSNs of NAc.

D’Ascenzo and colleagues have reported that animals receiving cocaine suffer from impaired synaptic plasticity resulting from NMDAR dysfunction, which is caused by a decrease in D-serine level (D-serine is a co-agonist of NMDA receptor) [23]. The association between this impairment and cocaine addiction has been found. It is worth mentioning that the same type of impairment has also been observed in some CNS diseases, such as AD, schizophrenia, and depression [23]. Therefore, further studies on synaptic plasticity dysfunction could be a starting point for the development of new treatment strategies not only for addiction, but also for some mental illnesses and neurodegenerative diseases.

Among the important mechanisms determining the development of addiction are epigenetic processes, which involve the modification of the DNA structure and binding with histone proteins [24]. The influence of environmental stimuli on the activation or suppression of transcription and, consequently, on changes in gene expression could occur in this way. The specific regions in the genome rich in histone H3.3 are believed to indicate DNA sequences of genes binding with transcription factors involved in the cocaine-induced plasticity (e.g., Npas4, Nptx2, Pmepa1, Vgf, and Fosb) [24]. Alterations to the expression of the relevant genes may induce changes in BDNF concentration [25,26], morphogenesis of dendrites and the dendritic spines, along with an increase in their density [27], and clustering of AMPARs in synapses [21]. The consequence of these processes could be behavioral reactions, such as cocaine-conditioned place preference, drug self-administration, cocaine-induced locomotor activity, and spatial memory disturbances [24]. Altogether, the data indicate the involvement of various mechanisms of cocaine action upon the NAc during the development of addiction. This should be considered while searching for effective treatments for cocaine addiction.

A promising area for further research seems to be epigenetic mechanisms influencing changes in gene expression related to the maturation of synaptic systems, increased effectiveness of synaptic transmission, and characteristics of receptors.

### 2.3. Morphine

Another addictive substance having considerable influence on the processes occurring in the NAc is morphine. The results of experimental studies have shown that chronic use of morphine contributes to the reduced expression of BDNF and TrkB receptors in the NAc (Figure 1) [28]. The coexistence of this phenomenon with decreased inhibition via GABAARs of D1R-MSNs may contribute to an enhancement of the reward feeling after morphine administration and to intensification of addiction.

Morphine affects the synaptic plasticity which determines the functioning of the BRS. Administration of morphine induces morphological changes leading to an increase in the dendritic spines’ density in the MSNs in the NAc [5]. This suggests the involvement of this opioid in the modification of the structural plasticity in this brain area. Interestingly, the way of morphine administration seems to be important for the dynamics of this process. In animals trained for self-administration, the increase in the dendritic spine density was higher than in those to whom it was administered regardless of their will [5]. Although the reason behind this difference has not been elucidated, it may suggest the induction of different signaling pathways depending on the route of administration of the substance, as well as the effect of the additional stimulation of the limbic system in the case of the forced morphine administration.

A long-term morphine administration causes changes in the DA metabolism in the basal ganglia which finally leads to a decrease in the level of this neurotransmitter in the NAc [29]. Interestingly, the effect of morphine can be attenuated by physical exercise. A recent study has shown alleviated cognitive and behavioral deficits caused by morphine use in female rats forced to physical exercise [30]. In conclusion, the presented data indicate a plethora of morphine functional and morphological effects exerted upon the NAc activity. However, a full explanation of its role in the regulation of the BRS functioning awaits further research.

### 2.4. Nicotine

Nicotine stimulates cholinergic receptors located on the dopaminergic projection neurons in the BRS. The long-term use of nicotine also increases the neuronal expression of NMDAR in the NAc (Figure 1). Interestingly, this effect has been reported only in the core [31]. This may be due to a stimulating effect, which contributes to the activation of signaling pathways and behavioral reactions aimed at achieving satisfaction and reward. Administration of nicotine increases levels of pCREB and DeltaFosB in the BRS areas [32]. Previously published results of studies on the nicotine-induced activation of transcription factors indicate a considerable diversity in this process among the various BRS structures [33,34,35]. For example, whereas nicotine induces a significant increase in the pCREB in the NAc, the DeltaFosB increase is more pronounced in the Amg. This can be explained, on the one hand, by the involvement of these areas in different aspects of behavioral reaction in response to the stimulus. On the other hand, this indicates the possibility of triggering different signaling pathways and regulatory mechanisms in various brain regions. A better understanding of the expression patterns of pCREB and DeltaFosB and the signaling pathways they are involved in under nicotine stimulation may be of importance for the development of new treatments for nicotine addiction.

Interestingly, changes in neuroplasticity within the BRS have also been observed in the case of nicotine withdrawal. Cessation of nicotine administration after chronic use has induced an increase in the BDNF level in the NAc and VTA neurons in mice [36]. This may suggest the occurrence of adaptation processes based on functional and structural plasticity changes. They could reduce the negative effects of nicotine withdrawal, but also support signaling pathways and behavioral reactions associated with the desire to restore the well-being status. In addition, a recent study found that a short-term 24-48 h nicotine withdrawal increased mRNA levels of NPY Y1 and Y2 receptors in the NAc [37]. This may suggest the participation of this neuropeptide in the development of negative affective symptoms initiated by nicotine withdrawal.

Nicotine is an example of a stimulating agent acting on the BRS in different ways. It induces the activation of several signaling pathways and participates in various aspects of behavioral reactions depending on the brain area. Interesting results can be obtained in the future by studying the signaling pathways involved in nicotine stimulation of the NAc, as well as changes in their activity during withdrawal.

## 3. Impairment of the Synaptic Plasticity and Dysregulation of Signaling Pathways Contribute to the Brain Reward System Dysfunction in Mental Illnesses

### 3.1. Depression

A low self-esteem accompanied by anxiety, loss of pleasure, appetite and sleeping disorders are among the most important symptoms of major depressive disorder (MDD) [38,39]. Apart from genetic predispositions and chronic stress contributing to its development, some evidence also suggests that structural and functional alterations in the BRS could be involved in the development of MDD. The important role in this process is played by a functional imbalance of the connections between the ventral tegmental area (VTA) and the NAc [40], as well as between the VTA and the medial prefrontal cortex (mPFC) [41,42,43]. Interestingly, while inhibition of interactions between the VTA and mPFC increases susceptibility to MDD, inhibition of interactions between the the VTA and NAc has been suggested to increase the resistance to depression [41,42,43].

Other studies demonstrated a relationship between a reduction in the volume of several brain regions (including the NAc) and the development of the MDD symptoms or attenuated reward response [44,45]. A correlation has been found between the co-existence of MDD with chronic stress and morphological changes to the structure of the neuronal dendritic tree in various brain regions [46]. However, these effects may vary depending on the patient’s age, the severity of the pathological process, the therapy introduced (or not), and the area of the brain studied [47,48]. While in Hip and PFC these changes include an atrophy and loss of dendritic spines, in the NAc and Amg the density of dendritic spines increases [46]. One possible explanation could be a disruption of the mTOR-dependent signaling pathway, which varies among brain regions. It has been present, for instance, in the PFC of patients with MDD [46]. Hence, a comparative assessment of morphological changes in the spino-dendritic system, as well as the expression level of mTOR in the BRS of patients with depression, could shed more light on the pathomechanism of this illness.

Importantly, up-regulation of mTOR induced by a stimulation of AMPARs and by an increase in BDNF level, especially in the Hip and PFC, underlies the antidepressant action of ketamine [46]. This induces an increase in the content of the synaptic signaling proteins, as well as an increase in the number of spinal synapses [49]. Further studies on the role of the mTOR-dependent pathways in MDD may be important for the design of new antidepressant drugs [50]. Studies have also shown that chronic stress can initiate atrophy and remodeling of the dendritic system in the D1R-MSNs in the NAc by activating the RhoA-dependent pathway [51,52]. This may result in a decreased D1R-MSNs-mediated synaptic stimulation, which can be manifested by anhedonia and followed by the development of MDD.

An important role in development of MDD has also been attributed to BDNF. Changes in the expression of this neurotrophic factor in the BRS are triggered by both MDD and stress [53]. When both are present, the BDNF-related signaling is reduced in the Hip and PFC, while it increases in the Amg and NAc (Figure 1) [54,55,56]. Patients with schizophrenia and comorbid depression have decreased levels of BDNF and TrkB in serum and in the Hip [57,58,59], which may be accompanied by a decrease in the hippocampal volume [60]. This, in turn, could have a significant impact on the course of both pathologies. The BDNF expression-related changes could also be responsible for the severity of anxiety and cognitive disorders.

Some reports suggest disturbances in the functioning of the neurotransmitter systems in the NAc as one of the causes of MDD [61]. They could be triggered directly by damage to the neuronal subpopulations in the NAc caused by local harmful factors. A recently formulated hypothesis suggests a link between chronic unpredictable mild stress (CUMS) along with MDD and dysfunction of the GABA-ergic and dopaminergic neurotransmitter systems in the NAc [61]. This could be a consequence of stress-induced autophagy and apoptosis. However, identification of the factors responsible for triggering death mechanisms in the course of MDD requires further studies. In this context, an important issue that may contribute to a better understanding of these mechanisms seems to be the attempt to link depressive-like behaviors with the reduction of dopaminergic D3R receptor expression, the activation of microglia in the NAc, and the development of neuroinflammation [62].

Another area of research on the molecular factors involved in the development of depression focuses on transcription factors. Among these, one of the most important is NF-ĸB. Its role may result from involvement in dysregulation of neurogenesis and synaptic transmission. It has been suggested that enhancement of MDD symptoms may be a consequence of the decrease in the BDNF level with a simultaneous increase in NF-ĸB [63]. In this context, a crucial issue seems to be keeping the dynamic balance between processes regulated by NF-ĸB and BDNF.

Taken together, the results published so far justify the need to continue research aimed at combining neurobiological observations (e.g., morphometric assessment of changes in the NAc structure, the efficiency of its connections) with the results of clinical studies on the nature and severity of behavioral manifestations of depression.

### 3.2. Schizophrenia

Characteristic structural and functional changes in the NAc and other areas of the BRS have been observed in patients with schizophrenia [64]. Considering the integrative function of the NAc in information transfer between the PFC and Hip, the cortico-limbic dissociation occurring in schizophrenia is largely a consequence of the NAc dysfunction [64]. Hyperactivity of the ventral subiculum (vSub), being a part of the hippocampal complex, has been shown in schizophrenia. Moreover, it corresponded to the severity of the presented positive symptoms [64]. It has been suggested that vSub hyperactivity impairs NAc function through its reciprocal connections [65,66,67]. This could explain the appearance and exacerbation of psychotic symptoms. In physiological conditions, the mPFC exerts most likely an inhibitory effect upon the vSub, which indirectly modulates NAc activity [68,69]. The role of the NAc in this system is the integration of contextual stimuli arising in the Hip with patterns of behavioral performance created in the mPFC. Additionally, in the NAc these stimuli are modulated by dopaminergic projection originating in the VTA. The physiological effect of this complex integration is control over behavioral patterns aimed at reward achievement [70,71]. An excessive activation of the vSub, as a result of PFC dysfunction in schizophrenia, changes modulation in the mPFC-vSub-NAc system. This results in development of pathological symptoms [68,69].

There is no consistent data on DA levels in the NAc in patients with schizophrenia. Two postmortem studies found an increased concentration of DA in this brain area [72,73]. However, the results of a more recent study suggest no changes in DA level [74].

Another suggested pathomechanism in schizophrenia is changes in the BDNF expression. However, data on this issue is still inconclusive [75,76]. Although some evidence indicates a decrease in BDNF in serum from patients with schizophrenia, other studies have shown its elevated level (Figure 1). This could be explained either by an unequal degree of brain damage in the analyzed cases or by the administration of various drugs affecting the BDNF expression. There is also a contradictory evidence for both the elevation and reduction of BDNF levels in the frontal cortex [77,78] and in the Hip in the course of schizophrenia [79,80]. A recently published clinical study has reported significantly lower BDNF plasma concentrations in schizophrenic patients with concomitant depression [81]. Although an explanation of this issue requires further studies, the consequences of it can be related with morphological changes affecting the spino-dendritic system, resulting in disruption of the morphological and functional plasticity, and aggravation of the clinical symptoms. A few studies reported a reduced volume of the PFC with the concomitant decrease in the BDNF content [82,83,84]. In other brain areas (e.g., the NAc and Amg), on the contrary, an increase in BDNF concentration [85] correlating with an increase in volume was reported [86,87]. Although explanation of these differences requires further research, one can speculate that the opposite tendencies in the volume changes observed in the various brain areas can result from the changes occurring in their neuropil (composed mainly of the axo-dendritic elements), initiated by the alterations in the plasticity processes. This could lead to the activation of signaling pathways involved in the initiation of different behavioral response patterns. The consequence of this, in turn, could be an aggravation of symptoms.

Recent studied have shown that in many mental diseases, including schizophrenia, an inflammatory process develops in NAc, and this is associated with abnormal DA concentration in this brain area [88,89]. This can result in neuronal atrophy and changes in the axo-dendritic system, leading to the exacerbation of disease symptoms. Mitigating the consequences of this inflammatory process requires further in vivo and clinical research.

Reassuming, among the most important functions of the NAc in the pathophysiology of schizophrenia one can include the excessive activation of the mPFC-vSub-NAc axis, unstable BDNF expression and, finally, changes in the NAc volume most probably resulting from neuropil modifications which influence neuroplasticity processes. These data suggests that the NAc is an important brain region involved in the pathogenesis of schizophrenia.

## 4. The Nucleus Accumbens Contribution to Neurodegeneration Results Not Only from Changes Occurring within the Nucleus Itself but Also within the Functionally Related Brain Areas

### 4.1. Alzheimer’s Disease

The role of the NAc in the pathogenesis of AD is a consequence of processes occurring in this brain region, as well as of the constantly changing influence of the other degenerated brain areas connected to the NAc. Although it is well known that in the course of AD there are volume changes of the limbic structures, only recently the atrophy of NAc has been reported as a consequence of this process [90]. The volume reduction has a regional character and occurs mainly in the ventral and posterior parts of NAc. Interestingly, the total volume of the nucleus remains unchanged. On the one hand, this could probably explain the lack of data on this phenomenon in previous studies. On the other hand, this suggests possible differentiation of metabolic and signaling processes occurring in different parts of the NAc. Furthermore, the volume reduction in different parts of the NAc could result in cognitive, motivation, pleasure, and reward disorders. The issue of internal molecular and functional differences between the various parts of the NAc needs further investigation. These studies may also be a good starting point for application of the deep brain stimulation (DBS) in AD therapy [90]. Indeed, previous reports suggested the possibility of the NAc ablation or stimulation as a treatment strategy for some CNS disorders [1].

Another important issue in the context of AD neuropathology is the accumulation of tau protein in the NAc (Figure 1). This has been reported in tangle predominant dementia (TPD), a late onset dementia showing similar features to AD and considered by some authors as its atypical form [91]. The pathomechanism of tau protein deposits’ formation in the NAc is still the subject of investigation. It has been suggested that their spread in the brain may be a consequence of expansion which occurs along the nerve fibers between the regions having strong connections [91]. According to this hypothesis, accumulation of the tau protein deposits in NAc is a consequence of their earlier appearance in the Hip (e.g., the CA1 sector and subiculum). Thus, the accumulation of tau protein may be indicative of the duration and severity of the pathological process. Importantly, the presence of tau protein in the NAc has also been observed in patients with AD [91].

An example of the influence of the neighboring structures on the NAc in AD is reduced dopaminergic projection, which results from a decrease in the amount of DA produced in the VTA. Studies on an animal model of AD (Tg2576 mice) have shown a decreased number of dopaminergic cells in the VTA due to their apoptotic death [92]. In consequence, dysregulated interactions between the VTA, Hip, and NAc were recorded [93]. This could lead to an impairment of memory, influence new and permanent memory traces formation, and affect the efficiency of spatial memory and reward processing.

Recently, a decrease in glycine receptors (GlyR) together with a reduction in the pre- and post-synaptic proteins SV2 and gephyrin, respectively, were reported in an animal AD model [94]. These observations could explain not only the nature of morphological synaptic changes, but also the decrease in the efficiency of synaptic transmission occurring in early stages of AD.

During AD progression, changes in BDNF expression have also been reported. In physiological conditions, this neurotrophic factor increases the chances for neuronal survival [95,96]. The action of BDNF is based on involvement in the synaptic plasticity via enhancement of the dendritic tree, axonal growth, and neurotransmitters’ release. In addition, the results of both in vitro and in vivo studies have shown the neuroprotective effect of BDNF reflected in decreased production and accumulation of amyloid ß (Aß) within the brain tissue [97,98]. This results in reduced neurotoxicity and the likelihood of cell death and synaptic dysfunction [99,100] as a result of synaptic insertion of calcium-permeable AMPA receptors normally absent in the NAc neurons [101]. Furthermore, by supporting the processes of synaptogenesis and synaptic plasticity, BDNF improves cognition, learning abilities, and memory consolidation [95,102]. This factor is also responsible for reduction of the spatial memory deficits [103,104]. These effects are consequences of the improvement of long-term synaptic transmission efficiency, initiated through the induction and maintenance of the LTP.

Several clinical reports have shown a decrease in BDNF mRNA and protein expression in the Hip and cerebral cortex in AD patients [105,106]. However, decrease in both the pro-BDNF and BDNF levels has been observed only at the early stages of disease, preceding a decrease in the choline acetyltransferase activity [107]. Moreover, a negative correlation between decreased BDNF and increased severity of cognitive impairment has been observed. Therefore, according to some experts, BDNF could be of potential diagnostic value as a biomarker for the early stage of AD [107].

Apart from the expression changes observed at the early phase of AD, the effects of BDNF disturbance were also manifested at later stages of this neurodegenerative disease [108]. They have been attributed to increased and accelerated accumulation of Aß and activation of the inflammatory response. The consequences of decrease in the BDNF content could be changes in the number and types of receptors, decrease in the number of synapses, weakening of the LTP and, consequently, reduction in the efficiency of synaptic transmission. All these changes could result in deterioration of the cognitive functions.

Hence, important aspects of potential NAc involvement in AD development and promising areas for further research include dysregulated VTA-Hip-NAc interactions, the NAc volume changes, tau protein accumulation, and decreased dopaminergic control of NAc activity.

### 4.2. Huntington’s Disease

NAc volume reduction, being a consequence of atrophy, has been observed in patients with HD. However, in this region of the ventral striatum, the atrophy is not as pronounced as in the dorsal striatum [109,110]. Nonetheless, this parameter may still have some value in assessing the disease progression. The morphological analysis of the NAc’s shape showed a correlation between the extent of atrophy and the disease progression during both the pre-manifestation and the manifestation periods [110]. In the latter, the changes were especially clearly presented in the caudal part of the NAc. This may be due to a differentiated internal structure of this brain region, its diversified function or heterogeneous susceptibility to neurodegeneration.

The neurodegenerative changes in the cellular structure of the NAc are diversified [111]. While the changes in the density of small neurons are lesser than in the dorsal striatum, the changes in the density of large neurons are more pronounced. Moreover, they also depend on the phenotype of the onset of the clinical symptoms. The density of the large neurons in the NAc is increased in patients with neuropsychiatric symptoms compared to patients with the motor symptoms [111].

In patients with HD, the atrophy of the NAc is not as pronounced as of the other parts of the basal ganglia [109]. The changes in the NAc’s cellular structure are characteristic and affect mainly the projection neurons and, to a lesser extent, the interneurons. Indeed, a few studies showed spared subpopulations of the medium-sized spiny neurons which contained NADPH-d, somatostatin (SOM) and neuropeptide Y (NPY), in the NAc of patients with HD [112,113]. Moreover, the cellular density of these neurons was even increased in the NAc (part of the ventral striatum) compared to the dorsal striatum. The elevated level of SOM in the NAc was accompanied by a higher concentration of SOM receptors in this brain region in patients with Huntington’s chorea [109,114,115]. Furthermore, the intrinsic Enk (enkephalin)-, SP (substance P)- and AChE (acetylcholinesterase) -positive neurons and fibers were also spared in the NAc [116].

Growing evidence indicates changes in concentration of the classical neurotransmitters in the course of HD [117,118,119]. Although there has been reported a decrease in GABA and Glu throughout the entire striatum, their level is different in its various parts, which corresponds to the development of the pathological changes [118]. The decrease is the highest in the caudate nucleus, slightly lower in the putamen, and the lowest in the NAc. Similarly, differentiated dynamics characterizes changes in DA and serotonin (5-HT) concentrations in HD striatum [117]. DA level in the dorsal striatum is reduced. Additionally, striatal degeneration of dopamine D1- and D2-containing neurons in HD patients is accompanied by a decrease in the protein levels of D1R and D2R (Figure 1) [120,121]. At the same time, the 5-HT level is elevated. However, concentrations of both neurotransmitters remain unchanged in the NAc. Based on assessment of the immunoreactivity and the enzyme activity of AChE, Hammond and Brimijoin have reported that the level of acetylcholine (ACh) in the NAc in the brains of HD patients does not change [119]. This points to significant difference between the changes observed in HD and AD, where significant decreases in both the enzyme activity and the immunoreactivity of AChE in the NAc were reported [119].

Studies suggest an important role of BDNF in HD. Couly et al. showed a decrease in BDNF concentration in striatum in HD model [122], and overexpression of this neurotrophic factor in other brain regions ameliorated HD phenotypes in mice [123]. On the one hand, the data suggests the uneven dynamics of the atrophy in different parts of the striatum, whereas on the other hand, this may indicate the unequal susceptibility of the different cell subpopulations to damage and the occurrence of different metabolic conditions. This may explain the various degree of neurodegenerative processes. In addition, induced by the pathological processes changes in the functioning of the various neurotransmitter systems may explain the occurrence of the HD-specific symptoms like motor dysfunctions, neuropsychological and cognitive disorders, as well as impairment of concentration of attention, learning and memory.

Although the molecular basis of neuropsychological processes such as depression in HD is only partially understood, recently published results suggest a role of Cyclin-dependent kinase 5 (Cdk5)–related signaling pathway in the NAc in this pathology [124]. Through the action of Cdk5 upon dopamine- and cAMP-regulated phosphoprotein 32 (DARPP-32) and the phosphorylation of β-adducin, formation of the dendritic spine cytoskeleton can be affected resulting in the development of depression.

Reassuming, a complex morphology along with characteristic cell populations, expression levels of certain neuropeptides, proteins and classical neurotransmitters, as well as signaling pathways in the NAc could explain the nature of pathological processes in HD. The link between the intensity of morphological changes in the NAc and the clinical picture in HD should be emphasized. Despite the fact that the intensity of changes in the NAc in HD is not as pronounced as in the other regions of the basal ganglia, further research on the NAc role in development of the neurodegenerative diseases is warranted. The obtained knowledge could prove to be useful for developing new diagnostic and therapeutic strategies against HD.

### 4.3. Parkinson Disease (PD)

A decrease in the NAc volume has also been observed in Parkinson’s disease [125,126,127]. In patients with PD, the percent of the NAc shrinkage is estimated for 11.77% [126]. One of the reasons at the base of these morphological changes is the reduced stimulating effect of DA resulting in the NAc’s neurons dysfunction, impairment of synaptic plasticity and, ultimately, decrease in the number of neurons [128]. A positive correlation between the decrease in the NAc volume and the intensity of its dysfunction has been shown. This involves both neuropsychiatric symptoms, such as depression, impairment of cognition and learning, apathy, anxiety, anhedonia [129], and motor dysfunction such as hypokinesia [128].

Furthermore, manifestation of the impulsive-compulsive behaviors in PD is related to the pathological changes in the NAc [130]. They could be a result of the decreased α-synuclein concentration and a reduction of the dopaminergic D3 receptors (D3Rs) in this brain area (Figure 1). Consequently, the control of the ventral striatum activity is reduced, which leads to its over-activity. It has been suggested that the reduced expression of D3Rs is caused by a reduction in BDNF levels, rather than a loss of DA [131].

In the course of PD, there are also disorders resulting from impairment of functional axes based on classic neurotransmitters, such as DA and Glu [132]. Although slight changes in their concentration are necessary for initiation of the goal-directed behaviors and motor learning, larger fluctuations can result in the development of addition and exacerbation of PD. The disturbance of the dopaminergic control of cortical, glutamatergic stimulation in the NAc can result in an impairment of motor activity learning, various forms of behavioral reactions, and in the development of addiction [132]. The reduced influence of the anterior cingulate cortex on the NAc lies at the base of the impulse-compulsive behaviors in PD patients [133].

Altogether, in patients with PD changes in the NAc are at the base of functional disorders associated with neuropsychological and motor dysfunctions. Due to the integrative role of the NAc, based on the interaction of several neurotransmitter systems, the spectrum of disorders resulting from its dysfunction is broad and often difficult to distinguish from impairment of the other basal ganglia. Hence, the role of the NAc in PD should be analyzed in close connection with the dysfunction of the other areas.

## 5. Conclusions and Perspectives for Future Research

This review summarizes the molecular and functional alterations which occur within the NAc in the course of several CNS pathologies, such as addiction, mental disorders (i.e., depression and schizophrenia), and neurodegenerative diseases (i.e., AD, HD, and PD). The role of the NAc in so many pathological processes can be explained based on its structural and functional characteristics. The NAc has extensive connections with the other brain regions organized in a hierarchal pattern that involves several neurotransmitter systems and well-developed synaptic plasticity mechanisms. This complex structural organization is exposed to the influence of pathological stimuli, of both external and internal origin, which are responsible for its disintegration. The morphological consequences induced by these factors are manifested by changes in the NAc’s volume, most likely resulting from changes in the neuropil volume and cell death, as well as the formation or disappearance of the dendritic tree elements, the dendritic spines, and synapses. The functional consequences involve changes in the synaptic plasticity, which lead to a decreased effectiveness of the neurotransmission. They also include changes in the expression of some receptors (e.g., NMDAR, AMPAR D1R, D2R) and the composition of their subunits. All these alterations can be induced by either external (e.g., addictive substances) or internal factors (e.g., metabolic disorders, dysregulation of signaling pathways or genetic conditions) and lead to dysfunction of the signaling pathways, changes in the content of neuromodulators (e.g., BDNF), transcription factors (e.g., pCREB, DeltaFosB, NFkB), histones (e.g., histone H3.3) and, ultimately, changes in the gene expression. Importantly, changes in the NAc have a different character and intensity compared to those observed in the other parts of the basal ganglia, in particular in the dorsal striatum, which emphasizes the functional differences between these two brain regions.

We propose that the role of NAc in the development of pathological processes should be seen in several aspects. First, one should consider the morphological changes of neurons and neuropil, together with dysfunction of the signaling pathways and the decreased effectiveness of the synaptic transmission, all of which could explain the nature of the pathological processes destroying the NAc itself. Secondly, changes in the NAc connections with the other brain areas involved in the specific pathologies should be taken into account. Thus, what happens within the NAc must be considered as a result of processes occurring simultaneously in many brain areas, related reciprocally to each other in the majority of cases. Thirdly, the pathological changes and the resulting signs and symptoms are, to a large extent, determined by the stage of the disease. Consequently, the NAc damage is not an isolated phenomenon that occurs independently from damage to the other brain regions. On the contrary, this is an effect of processes acting simultaneously in many brain regions and functional systems, although the nature of these destructive processes and their intensity may considerably differ among them.

Future research on the role of NAc in the course of various pathological processes should focus on several issues. First, valuable information can be obtained from the broadly understood morphological studies of this nucleus focused on the assessment of changes in its volume, cellular and neuropil content, as well as morphological changes occurring within its parts, which has not been described in detail so far. Second, research on NAc connections with other brain regions, in particular those constituting the limbic system, seems to be very promising. The results of these studies will contribute to a better understanding of the pathomechanisms of addictions development, mental disorders, and neurodegenerative diseases. Third, a valuable complementation to these studies may be the assessment of changes occurring in the NAc related to structural and functional synaptic plasticity disorders, changes in the neurotransmitters’ and neuromodulators’ concentrations, the structure and number of receptors and, consequently, changes in the effectiveness of synaptic transmission. Fourth, studies on changes in gene expression and epigenetic modifications and in the effectiveness of signaling pathways in the course of CNS diseases can provide valuable information. Finally, it should be emphasized that a comprehensive understanding of the role of NAc in a specific CNS pathology requires a translational approach that combines the results of basic and clinical research and takes into consideration the coexistence of comorbid pathological processes. We believe that the presented view on the changes occurring in NAc, and resulting from them pathological symptoms, can be useful not only for setting new directions of research but also in designing new diagnostic and therapeutic strategies against the CNS diseases.

## Data Availability

Not applicable.

## Abbreviations

Aß	amyloid ß
ACh	acetylcholine
AChE	acetylcholinesterase
AD	Alzheimer’s disease
Amg	amygdala
AMPARs	α-amino-3-hydroxy-5-methyl-4-isoxazolepropionic acid-type glutamate receptors
BDNF	brain-derived neurotrophic factor
BRS	brain reward system
Cdk5	cyclin-dependent kinase 5
CNS	central nervous system
CUMS	chronic unpredictable mild stress
DA	dopamine
DARPP-32	cAMP-regulated phosphoprotein 32
DBS	deep brain stimulation
D1R	D1 dopamine receptor
GABA	γ-aminobutyric acid
GABAARs	γ-aminobutyric acid A receptors
Glu	glutamate
GluD1R	glutamate delta-1 receptor
HD	Huntington’s disease
Hip	hippocampus
5-HT	serotonin
LTP	long-term potentiation
MDD	major depressive disorder
MGluRs	metabotropic glutamatergic receptors
MSNs	medium spiny neurons
mPFC	medial prefrontal cortex
NAc	nucleus accumbens
NADPH-d	dihydronicotinamide adenine dinucleotide phosphate diaphorase
NMDARs	N-methyl-D-aspartate receptors
NPY	neuropeptide Y
pCREB	phosphorylated cAMP (adenosine 3′5′ cyclic monophosphate)-Response Element Binding protein
PD	Parkinson’s disease
PFC	prefrontal cortex
SOM	somatostatin
TPD	tangle-predominant dementia
TrkB	tropomyosin receptor kinase B
vSub	ventral subiculum
VTA	ventral tegmental area
Enk	enkephalin
SP	substance P
